# Complementary Critical Functions of *Zfy1* and *Zfy2* in Mouse Spermatogenesis and Reproduction

**DOI:** 10.1371/journal.pgen.1006578

**Published:** 2017-01-23

**Authors:** Takashi Nakasuji, Narumi Ogonuki, Tomoki Chiba, Tomomi Kato, Kumiko Shiozawa, Kenji Yamatoya, Hiromitsu Tanaka, Tadashi Kondo, Kenji Miyado, Naoyuki Miyasaka, Toshiro Kubota, Atsuo Ogura, Hiroshi Asahara

**Affiliations:** 1 Comprehensive Reproductive Medicine, Regulation of Internal Environment and Reproduction, Systemic Organ Regulation, Graduate School, Tokyo Medical and Dental University (TMDU), Bunkyo, Tokyo, Japan; 2 Department of Systems BioMedicine, Tokyo Medical and Dental University (TMDU), Bunkyo, Tokyo, Japan; 3 RIKEN BioResource Center, Tsukuba, Ibaraki, Japan; 4 Division of Rare Cancer Research, National Cancer Center Research Institute, Chuo, Tokyo, Japan; 5 Department of Reproductive Biology, National Center for Child Health and Development, Setagaya, Tokyo, Japan; 6 Molecular Biology Division, Faculty of Pharmaceutical Sciences, Nagasaki International University, Sasebo, Nagasaki, Japan; 7 Department of Molecular and Experimental Medicine, The Scripps Research Institute, La Jolla, California, United States of America; University of Nevada School of Medicine, UNITED STATES

## Abstract

The mammalian Y chromosome plays a critical role in spermatogenesis. However, the exact functions of each gene in the Y chromosome have not been completely elucidated, partly owing to difficulties in gene targeting analysis of the Y chromosome. *Zfy* was first proposed to be a sex determination factor, but its function in spermatogenesis has been recently elucidated. Nevertheless, *Zfy* gene targeting analysis has not been performed thus far. Here, we adopted the highly efficient CRISPR/Cas9 system to generate individual *Zfy1* or *Zfy2* knockout (KO) mice and *Zfy1* and *Zfy2* double knockout (*Zfy1/2*-DKO) mice. While individual *Zfy1* or *Zfy2-*KO mice did not show any significant phenotypic alterations in fertility, *Zfy1/2-*DKO mice were infertile and displayed abnormal sperm morphology, fertilization failure, and early embryonic development failure. Mass spectrometric screening, followed by confirmation with western blot analysis, showed that PLCZ1, PLCD4, PRSS21, and HTT protein expression were significantly deceased in spermatozoa of *Zfy1/2-*DKO mice compared with those of wild-type mice. These results are consistent with the phenotypic changes seen in the double-mutant mice. Collectively, our strategy and findings revealed that *Zfy1* and *Zfy2* have redundant functions in spermatogenesis, facilitating a better understanding of fertilization failure and early embryonic development failure.

## Author Summary

The Y chromosome is known to play a critical role in spermatogenesis based on human genetic evidence. However, specific Y chromosome genes responsible for spermatogenesis have not been fully elucidated yet, because the targeted deletion of Y chromosome genes is difficult owing to its unique structure. In this study, using our highly optimized CRISPR/Cas9 system, we disrupted *Zfy1* and *Zfy2* ampliconic genes located on the Y chromosome in double-deletion mutant mice. By analyzing the mice, we revealed that *Zfy1* and *Zfy2* have redundant functions, and that a lack of both *Zfy1* and *Zfy2* leads to male infertility. *Zfy1* and *Zfy2* double-deletion mutant mice showed severe abnormalities in their sperm, including defects in morphology, motility, capacitation, acrosome reaction, and oocyte activation, as well as chromosomal aberrations, indicating that *Zfy1* and *Zfy2* are required for multiple aspects of spermatogenesis. Elucidating the mechanisms that underlie *Zfy*-dependent spermatogenesis will shed light on the failure of fertilization and early embryonic development in mammals.

## Introduction

The Y chromosome plays an important role in spermatogenesis and sex determination [1,2]. However, specific Y chromosome genes responsible for spermatogenesis and sex determination have not been determined in mice using gene-targeting approaches because of challenges associated with genome recombination in the unique structure of the Y chromosome [3]. Therefore, transgene strategies using female mice or mice with multiple Y chromosome gene deletion variants have been performed [4,5]. However, the physiological role of genes encoded on the Y chromosome remains unclear, particularly in relation to genes responsible for infertility.

Artificial endonuclease methods such as TALEN (transcription activator-like effector nuclease) and CRISPR/Cas9 (clustered regularly interspaced short palindromic repeat/CRISPR-associated 9) systems have been widely used for genome editing. The CRISPR/Cas9 system originates from the adaptive immunity of bacteria to viruses and plasmids, and works by introducing a site-specific double-strand break in the DNA [6]. The CRISPR/Cas9 system has been used for producing knockouts in the male germline [7–9]. We and other researchers have successfully applied the TALEN or CRISPR/Cas9 systems to Y chromosomal gene targeting and have confirmed the key functions of *Sry* in sex determination and *Eif2s3y* in spermatogonial stem cell development [3,10–12].

The two homologous *Zfy* genes, *Zfy1* and *Zfy2*, are located on the short arm of the Y chromosome, and *Zfy* was initially proposed to be a sex-determining factor [13–15]. However, later studies demonstrated that another Y chromosome gene, *Sry*, was responsible for sex determination, and the function of *Zfy* has thus been unclear for a long time [4,16,17]. *Zfy1* and *Zfy2* are considered to be transcription factors and are thought to be involved in transcriptional activation because they encode proteins possessing both zinc finger domains and an acidic domain, which is known to be involved in transcriptional activation [18,19]. Recent studies have shown that *Zfy1* and *Zfy2* play an important role in spermatogenesis, particularly in promoting meiotic division [20] and sperm formation [21,22]. However, there are no data about mice deficient in either individual or both *Zfy* genes, and therefore, the exact physiological functions of *Zfy1* and *Zfy2* are still unknown. In this study, we generated *Zfy1*-knockout (KO) mice, *Zfy2*-KO mice, and *Zfy1* and *Zfy2* double knockout (*Zfy1/2*-DKO) mice using the CRISPR/Cas9 system to examine the physiological roles of *Zfy1* and *Zfy2 in vivo*.

## Results

### Efficiency of generation of *Zfy1*-KO, *Zfy2*-KO, and *Zfy1/2*-DKO mice

**Fig 1:**
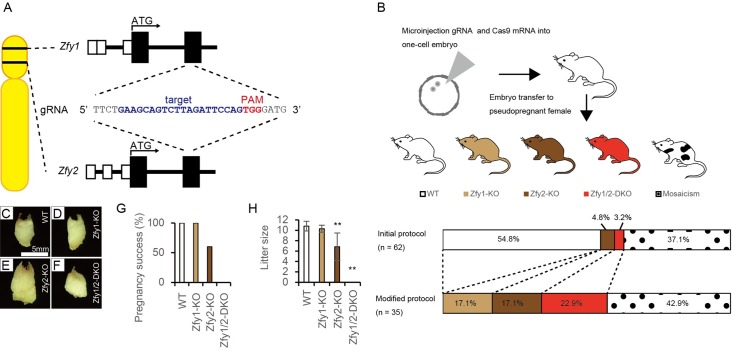
Strategy for and efficiency of generation of *Zfy1* knockout (KO) mice, *Zfy2*-KO mice, and *Zfy1* and *Zfy2* double knockout (*Zfy1/2*-DKO) mice, and the corresponding fertility of the mice. (A) Target sequence of *Zfy1* and *Zfy2* for guide RNA (gRNA). PAM; proto-spacer adjacent motif. (B) Strategy for microinjection and embryo transfer, and efficiency of generation of mutant mice. (C to F) Vaginal plug of WT (C), *Zfy1*-KO (D), *Zfy2*-KO (E), and *Zfy1/2*-DKO (F). (G) Pregnancy success rates of WT, *Zfy1*-KO, *Zfy*2-KO, and *Zfy*1/2-DKO mice were 100%, 100%, 60%, and 0%, respectively. (H) Average litter sizes of WT, *Zfy1*-KO, *Zfy*2-KO, and *Zfy*1/2-DKO mice were 10.7, 10.3, 7, and 0, respectively. Error bars; S.D., n = 10, ** *p* < 0.01 compared with WT (Student’s *t*-test).

To generate *Zfy1*-KO, *Zfy2*-KO, and *Zfy1/2*-DKO mice, the CRISPR/Cas9 system was further optimized from our initial protocol [11]. A guide RNA (gRNA) was designed to target the common sequences in exon 4 of both the *Zfy1* and *Zfy2* genes, which are located downstream of the second methionine (Fig 1A). This should prevent potential expression of the functional variant from the second methionine. The gRNA and Cas9 mRNA were microinjected into one-cell embryos, which were transferred to pseudopregnant female mice. To validate nucleotide insertion or deletion at the target loci, corresponding regions of both *Zfy1* and *Zfy2* were amplified by PCR and sequenced. As the efficiency of generation of *Zfy1/2*-DKO mice was very low in the initial protocol, we made the time of microinjection earlier in the modified protocol (Fig 1B). In the modified protocol, all of male pups carried some mutations on *Zfy1* or *Zfy2*, and 22.8% of male pups had frameshift mutations on both *Zfy1* and *Zfy2*, all of which were introduced by the gRNA (Fig 1B). Sequences of the frameshift mutations are shown in S1 and S2 Figs.

### *Zfy2*-KO mice were subfertile and *Zfy1/2*-DKO mice were infertile

All *Zfy1*-KO, *Zfy2*-KO, and *Zfy1/2*-DKO mice were viable and showed normal development. Next, the fertility of these mice was examined by mating the mutant mice with fertility-proven (C57BL/6 x DBA/2) F1 (BDF1) female mice. Sexual behavior and vaginal plug formation were normal for all *Zfy1*-KO, *Zfy2*-KO, and *Zfy1/2*-DKO mice (Fig 1C–1F). Pregnancy success rates and average litter size did not differ between the wild-type (WT) male mice and *Zfy1*-KO male mice. Pregnancy success rates and average litter size were decreased in *Zfy2*-KO male mice (60% and 7, respectively) compared to WT male mice (100% and 10.7, respectively) (Fig 1G and 1H). Offspring of both mice grew normally and were fertile. All *Zfy1* or *Zfy2* mosaic mice were also fertile. In contrast, none of the 10 *Zfy1/2*-DKO male mice produced any pups after mating (Fig 1G and 1H), indicating that *Zfy1/2*-DKO male mice were infertile. We analyzed the *Zfy1/2*-DKO founder mice and offspring from *Zfy1*-KO and *Zfy2*-KO mice in the following experiments. To eliminate potential off-target effects of CRISPR/Cas9, three putative off-target sites of the gRNA were computationally predicted [23], amplified by PCR, and directly sequenced. None of the 10 *Zfy1/2*-DKO (#1–10) mice possessed a mutation in the three putative off-target sites (S1).

#### Defects in sperm morphology and motility in *Zfy1/2*-DKO mice

To investigate the age-related expression patterns of *Zfy1* and *Zfy2*, we performed quantitative real-time PCR of the testes of BDF1 mice ranging from 1 to 5 weeks old. The expression of *Zfy1* and *Zfy2* was elevated from that of 3-week-old mice (S3 Fig). These results suggest that *Zfy1* and *Zfy2* were expressed in the spermatocyte or spermatid.

**Fig 2:**
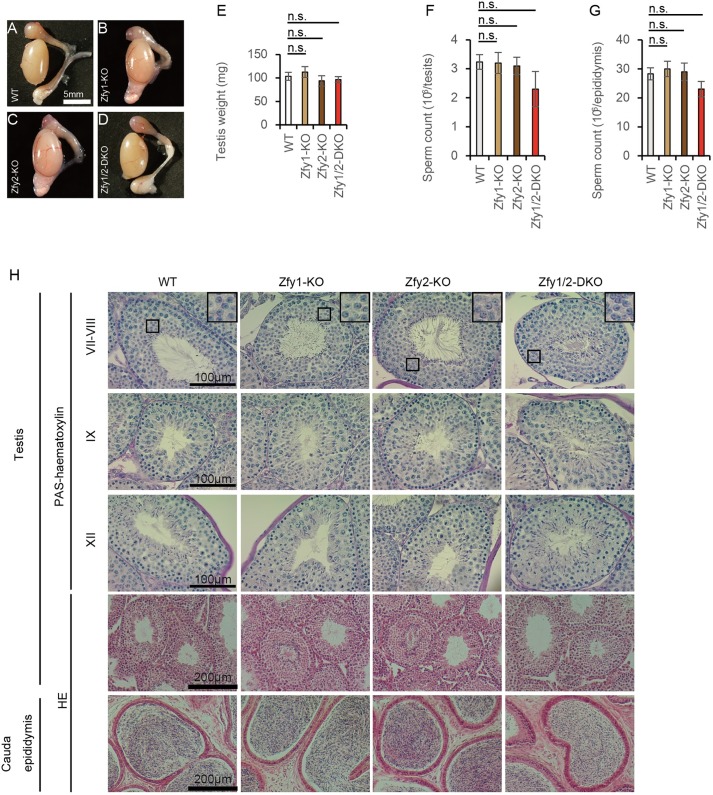
Histology analysis of testis and epididymis. (A to D) Gross appearance of the testis and epididymis. WT (A), *Zfy-1* knockout (KO) (B), *Zfy2*-KO (C), *Zfy1* and *Zfy2* double knockout (*Zfy1/2*-DKO) (D). (E) Testis weight of WT, *Zfy1*-KO and 2-KO, and 1/2-DKO mice. Error bars: S.D., n = 3. n.s.: not significant. (F) Sperm counts in the testis. Error bars: S.D., n = 3. (G) Sperm counts in the epididymis. Error bars: S.D., n = 3. (H) HE and periodic acid-Schiff (PAS)-haematoxylin staining of the testis and the cauda epididymis section of WT, *Zfy1*-KO, *Zfy2*-KO, and *Zfy1/2*-DKO mice. Roman numerals indicate the stages of the seminiferous tubules.

To investigate the cause of the abnormal fertility, we examined the gross appearance, sperm count, and histology of the testes and epididymides of adult mice after expression of *Zfy1* and *Zfy2* had begun. The testes and epididymides for all *Zfy1*-KO, *Zfy2*-KO, and *Zfy1/2*-DKO mice appeared grossly normal compared with those of WT mice (Fig 2A–2D), and the testis weights of all *Zfy1*-KO, *Zfy2*-KO, and *Zfy1/2*-DKO mice did not significantly differ from those of WT mice (Fig 2E). Testis and epididymal sperm counts did not significantly differ between WT, *Zfy1*-KO, *Zfy2*-KO, and *Zfy1/2*-DKO mice (Fig 2F and 2G). HE and periodic acid-Schiff (PAS)-haematoxylin staining results of testis tissue from all *Zfy1*-KO, *Zfy2*-KO, and *Zfy1/2*-DKO mice were also normal compared to those of WT mice (Fig 2H). HE staining of the epididymal tissue revealed lumina filled with sperm in *Zfy1*-KO, *Zfy2*-KO, and *Zfy1/2*-DKO mice (Fig 2H).

**Fig 3:**
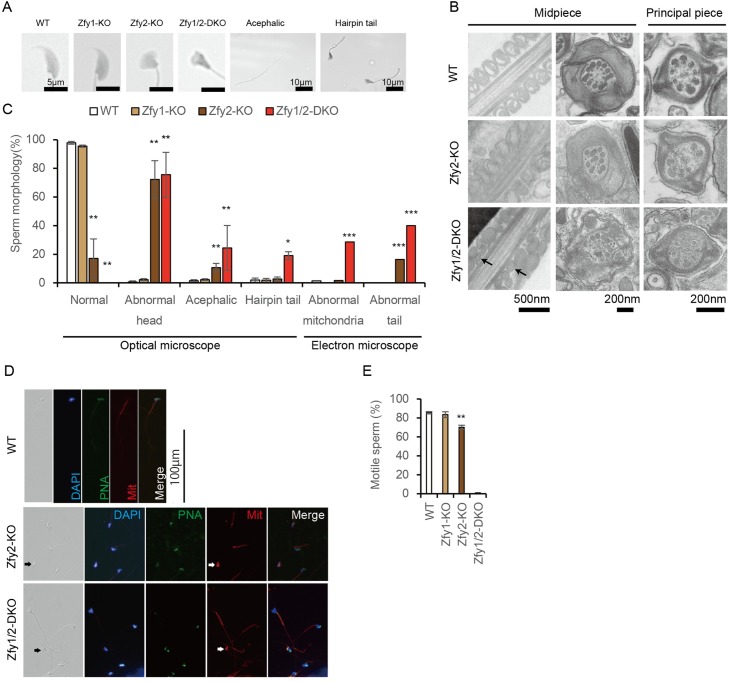
Histology and motility analysis of sperm. (A) Morphology of sperm in WT, *Zfy1* knockout (KO), *Zfy2*-KO, and *Zfy1* and *Zfy2* double knockout (*Zfy1/2*-DKO). (B) Transmission electron microscope analysis of WT, *Zfy2*-KO, and *Zfy1/2*-DKO. Arrows: abnormal mitochondria. (C) Summary of sperm morphology. Sperm were counted as more than 100 using an optical microscope. Error bars: S.D., n *≥* 3, * *p* < 0.05, ** *p* < 0.01 compared with WT (Student’s *t*-test). Sperm were counted as more than 50 using an electron microscope (*Zfy1*-KO; no data). n = 1, *** *p* < 0.01 compared with WT (two-tailed Fisher’s exact test). (D) Immunostaining of sperm. Epididymal sperm were separated on the glass slide and examined by fluorescent microscopy. Histological observations showed morphological abnormalities of mutant sperm. Formation of the nucleus, acrosome, and mitochondrion of the sperm were examined with DAPI, peanut agglutinin (PNA), and MitoTracker (Mit), respectively. Arrows indicated abnormal mitochondria. (E) Sperm motility was analyzed using computer-assisted sperm analysis. The ratios of motile sperm in WT, *Zfy1-KO*, *Zfy2*-KO, and *Zfy1/2*-DKO mice were 85.6%, 83.5%, 70.0%, 1.0%, respectively. Error bars; S.E.M. (WT, *Zfy1*-KO, and *Zfy2*-KO; n = 4, *Zfy1/2*-DKO; n = 1). ** *p* < 0.01 compared with WT (Student’s *t*-test).

Next, we examined sperm morphology and motility. The morphology of *Zfy1*-KO epididymal sperm was similar to that of WT (Fig 3A). However, morphologically abnormal sperm heads and acephalic sperm were clearly observed in the *Zfy2*-KO and *Zfy1/2*-DKO epididymis (Fig 3A). Hairpin tails were observed only in *Zfy1/2*-DKO sperm (Fig 3A). Transmission electron microscopic analysis revealed that *Zfy1/2*-DKO sperm have abnormal mitochondria and that *Zfy2*-KO and *Zfy1/2*-DKO sperm have disrupted axonemes and outer dense fibers (Fig 3B). Normal sperm were rarely observed in *Zfy2*-KO mice and were not observed in *Zfy1/2*-DKO mice (Fig 3C). Since morphologically abnormal sperm were observed in *Zfy2*-KO and *Zfy1/2*-DKO mice, we performed immunostaining of these sperm. Morphologically abnormal sperm heads, including acrosomes indicated by peanut agglutinin (PNA) and abnormalities of mitochondrial formation, were observed in *Zfy2*-KO and *Zfy1/2*-DKO sperm (Fig 3D). MCA, ODF3, and TEKT2, which are involved in flagellum formation, were observed in *Zfy2*-KO and *Zfy1/2*-DKO sperm (S4A Fig). Sperm nuclear proteins PRM1 and PRM2 were observed in *Zfy2*-KO and *Zfy1/2*-DKO sperm (S4B Fig). Next, we performed immunostaining of the testes. Formation of the nucleus, acrosome, flagellum, and mitochondrion in the testes was examined with DAPI, PNA, MCA, and MitoTracker, respectively. Spermiogenesis in the testes of WT, *Zfy2*-KO, and *Zfy1/2*-DKO showed no differences between these groups (S4C Fig). The results suggest that these structural proteins are expressed at the appropriate stage and in sufficient amounts to support sperm formation. Using computer-assisted sperm analysis, a reduced number of motile sperm was observed among *Zfy2*-KO sperm (70.0%) compared to WT sperm (85.6%). In addition, only 1.0% of sperm were motile in *Zfy1/2*-DKO mice (Fig 3E). These results suggest that the *Zfy* genes are involved in spermatogenesis and functionally compensate for each other.

### Characterization of *Zfy1/2*-DKO sperm *in vitro*

**Fig 4:**
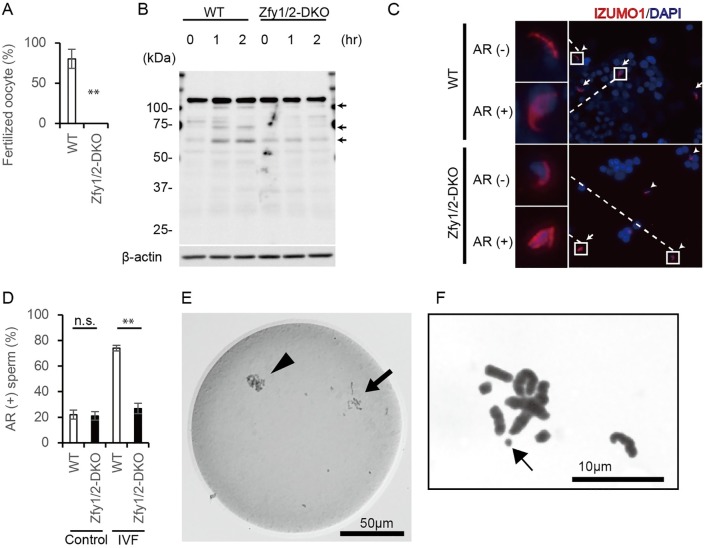
Characterization of sperm *in vitro*. (A) Ratio of fertilized oocytes by *in vitro* fertilization (IVF) using WT and *Zfy1* and *Zfy2* double knockout (*Zfy1/2*-DKO) sperm. n = 3, ** *p* < 0.01 compared with WT (Student’s *t*-test). (B) Tyrosine phosphorylation of sperm from cauda epididymis. Signals appeared to increase during incubation in the WT sperm (arrow), but not in the *Zfy1/2*-DKO sperm. (C) IZUMO1 localization after IVF, arrow; acrosome reacted sperm, arrow head; acrosome unreacted sperm. AR; acrosome reaction. (D) Ratio between spontaneous and IVF-induced acrosome reaction. n = 2, ** *p* < 0.01 compared with WT (Student’s *t*-test). (E) Premature chromosome condensation (arrow) observed in *Zfy1/2*-DKO sperm ICSI-derived embryo. Arrow head, oocyte chromosomes. (F) Chromosomal aberrations of intracytoplasmic sperm injection-derived mitotic embryos with *Zfy1/2*-DKO sperm. Arrow, chromosome break.

*Zfy1/2*-DKO mice were infertile and had abnormal sperm, but they did have a few motile sperm, so we performed an *in vitro* fertilization (IVF) assay with oocytes prepared from superovulated BDF1 female mice. However, *Zfy1/2*-DKO sperm failed to fertilize the oocytes *in vitro* (Fig 4A).

To investigate the characterization of *Zfy1/2*-DKO sperm *in vitro*, we examined sperm capacitation by global tyrosine phosphorylation state and acrosome reaction by IZUMO localization in WT and *Zfy1/2*-DKO sperm.

In order to induce sperm capacitation, sperm were incubated with HTF medium for 2 h. Western blot analysis revealed that tyrosine phosphorylation of WT sperm protein increased during 2-h incubation, but tyrosine phosphorylation of *Zfy1/2*-DKO sperm protein did not increase during 2-h incubation (Fig 4B). This result suggests that *Zfy* genes may regulate sperm capacitation.

Because capacitated sperm can undergo an acrosome reaction [24], we measured the acrosome reaction by IZUMO localization [25]. Sperm were incubated with control HTF medium or cumulus oocyte complexes and then stained with anti-IZUMO antibody. During the normal acrosome reaction, IZUMO relocates from the acrosomal cap to the equatorial and whole head regions [26]. IZUMO relocation did not differ between WT and *Zfy1/2*-DKO sperm using the control HTF medium, but it was significantly decreased in *Zfy1/2*-DKO sperm as compared to WT sperm after incubation with the cumulus oocyte complexes (Fig 4C and 4D).

These results suggest that the acrosome reaction was prevented in *Zfy1/2*-DKO sperm, partly due to a global absence of capacitation.

### *Zfy1/2*-DKO sperm failed to activate oocytes in ICSI and had chromosomal aberrations

To examine whether sperms from *Zfy1/2*-DKO mice retain oocyte activation ability and support subsequent embryonic development, we performed intracytoplasmic sperm injections (ICSI) with *Zfy1/2*-DKO sperm and BDF1 oocytes. Twenty-four hours after ICSI, the ratio of two-cell embryos was significantly lower in the *Zfy1/2*-DKO sperm group as compared to the WT group. In the *Zfy1/2*-DKO sperm-injected oocytes that failed to develop to the two-cell stage, premature chromosome condensation (PCC) was observed by whole-mount chromosomal staining (Table 1, Fig 4E). These results suggest that *Zfy1/2*-DKO sperm also exhibited reduced oocyte activation characteristics.

**Table 1:** Development of ICSI-derived embryos *in vitro*.

	No. of oocytes that survived injection	No. (%) at 24 h				No. (%) blastocyst at 96 h
		1-cell PN (-)	1-cell PN (+)	2-cell	Fragmentation	
WT	45	0 (0)	0 (0)	45 (100)	0 (0)	12/15 (80)
Zfy1/2-DKO #1	90	10 (11)*	26 (29)	52 (58)†	2 (2)	3/12 (13)†
Zfy1/2-DKO #2	85	57 (67)**	10 (12)	10 (12)†	8 (9)	N.D.

*Includes 9 with premature chromosome condensation.

**Includes 21with premature chromosome condensation.

† *p* < 0.01 compared with WT (two-tailed Fisher’s exact test).

PN: pronucleus, N.D.: no data (all embryos were transferred at the two-cell stage).

The ratio of blastocyst formation of ICSI-derived embryos was remarkably lower in the *Zfy1/2*-DKO sperm group compared to the WT sperm (Table 1). Furthermore, 10 embryo transfers of ICSI-derived embryos at the two-cell stage to pseudopregnant female mice did not generate any implantation sites and consequently resulted in no pups from the *Zfy1/2*-DKO sperm group.

To investigate whether chromosomal aberrations in *Zfy1/2*-DKO sperm could be the cause of the early failure in embryonic development, we performed chromosome analysis on the mitotic embryos derived from ICSI. Polyploidy (an excessive number of chromosomes) was not observed in *Zfy1/2*-DKO sperm-derived embryos (S5 Fig), but chromosome breaks were seen (Fig 4F). The frequency of chromosome aberrations at the first mitosis in WT and *Zfy1/2*-DKO was 0/20 (0%) and 3/15 (20%), respectively. This indicates that *Zfy* affects not only fertilization, but also the early embryonic developmental process associated with chromosome aberrations.

### Microarray and proteomic analysis

As *Zfy1* and *Zfy2* are considered to be involved in transcriptional activation, we performed microarray analysis of the WT and *Zfy1/2*-DKO testis and identified 1168 probe sets that had lower signals (<0.75) in *Zfy1/2*-DKO testis compared to WT testis (S2 Table) (GEO accession number: GSE90103). Functional annotation clustering among downregulated probe sets using the Database for Annotation, Visualization and Integrated Discovery (DAVID)-identified enriched GO terms that are related to reproduction, i.e., GO:0019953~sexual reproduction (P = 1.75E-04), GO:0048232~male gamete generation (P = 4.08E-04), GO:0007283~spermatogenesis (P = 4.08E-04), GO:0007276~gamete generation (P = 4.71E-03), and GO:0009566~fertilization (P = 4.82 E-03) (S3 Table). It also identified enriched GO terms that are related to phosphorylation, i.e., GO:0006796~phosphate metabolic process (P = 9.50E-3) and GO:0006793~phosphorus metabolic process (P = 9.50E-3) (S3 Table). These results suggest that *Zfy1* and *Zfy2* might regulate a set of genes involved in reproduction.

As mature sperms are transcriptionally and translationally silent [27], comprehensive liquid chromatography-tandem mass spectrometry analysis was used to elucidate the mechanisms underlying the abnormal sperm functions in *Zfy1/2*-DKO mice. Label-free proteomic analysis identified 3191 proteins in WT sperm. Among them, 1476 proteins were downregulated in *Zfy1/2*-DKO sperm to less than half the level of WT sperm (S4–S6 Tables) (ProteomeXchange Accession: PXD005438). Functional annotation clustering among downregulated proteins using DAVID identified enriched GO terms related to reproduction, i.e., GO:0019953~sexual reproduction (P = 1.52E-09), GO:0007338~single fertilization (P = 2.89E-07), GO:0009566~fertilization (P = 4.25E-07), GO:0048232~male gamete generation (P = 7.99E-06), and GO:0007283~spermatogenesis (P = 7.99E-06) (S7 Table). It also identified enriched GO terms related to phosphorylation, i.e., GO:0006793~phosphorus metabolic process (P = 1.88E-09), GO:0006796~phosphate metabolic process (P = 1.88E-09), GO:0016310~phosphorylation (P = 7.56E-08), GO:0006468~protein amino acid phosphorylation (P = 4.97E-07) (S7 Table).

**Fig 5:**
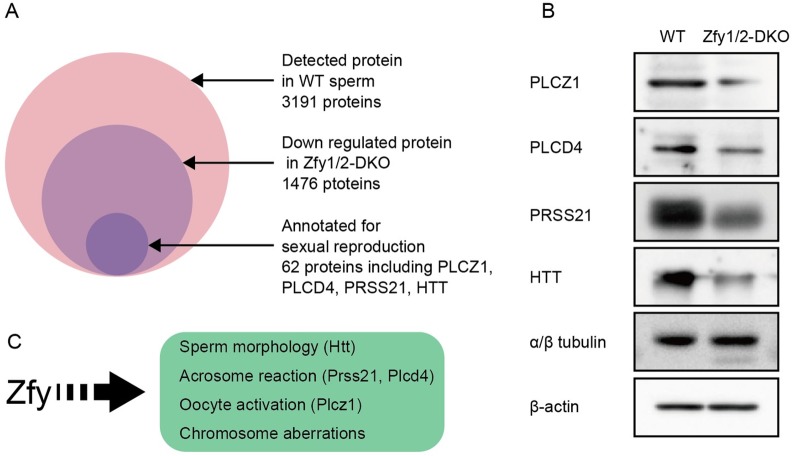
Sperm proteins downregulated in *Zfy1* and *Zfy2* double knockout (*Zfy1/2*-DKO) mice. (A) Mass spectrometric protein profiling of WT and *Zfy1/2*-DKO sperm. (B) Western blot analysis of sperm proteins extracted from cauda epididymis. α/β tubulin and β-actin were used as internal controls. (C) Scheme of proposed function of *Zfy*.

Functional annotation clustering analysis showed that 62 proteins were annotated with sexual reproduction (S8 Table). Of these, genes corresponding to the following phenotypes were present: PLCZ1 for oocyte activation [28], PLCD4 and PRSS21 for the acrosome reaction [29,30], and HTT for sperm head maturation [31] (Fig 5A). Western blot analysis confirmed that these four proteins were decreased in *Zfy1/2*-DKO sperm compared to in WT sperm (n = 2) (Fig 5B).

## Discussion

The Y chromosome has unique structural features, such as an ampliconic sequence and similarity to the X chromosome, and conventional gene targeting strategies using embryonic stem cells have been unsuccessful [3]. Furthermore, it is also difficult to disrupt simultaneously contiguous genes on the same chromosome. We previously used TALEN to generate individual *Sry-* and *Eif2s3y-*deficient mice, and demonstrated that *Sry* and *Eif2s3y* are prerequisites for sex determination and spermatogonial stem cell development, respectively [10,11]. In the present study, using our optimized CRISPR/Cas9 system, we clearly show that *Zfy1* and *Zfy2*, ampliconic genes located on the Y chromosome, have redundant functions in spermatogenesis and that lacking both *Zfy1* and *Zfy2* leads to male infertility due to abnormal sperm generation. Many KO mice have been generated with mutations in autosomal genes that are expressed in the testes, and have contributed to the analysis of gene function in male fertility [26]. Here, we provide novel insights into the roles of *Zfy1* and *Zfy2* in spermiogenesis.

*Zfy1/2*-DKO mice showed severe abnormalities in their sperm, including defects in morphology, motility, capacitation, acrosome reaction, and oocyte activation, as well as chromosomal aberrations, indicating that *Zfy1* and *Zfy2* are required at multiple aspects of spermatogenesis (Fig 5C). A previous study using transgenic mice suggested that *Zfy1* and *Zfy2* play critical roles in the second meiotic division [20]. In contrast, chromosomal analysis of ICSI-derived embryos did not show polyploidy, indicating that sperm in the epididymis of *Zfy1/2*-DKO mice completed meiotic division, and therefore, *Zfy1* and *Zfy2* could play essential roles in the later stages of spermatogenesis. Two previous studies reported that *Zfy2* is essential for sperm head and tail formation [21,22]. Among *Zfy1*-KO, *Zfy2*-KO, and *Zfy1/2*-DKO sperm, morphologically abnormal sperm were observed at 4.5%, 82.9%, and 100%, respectively (Fig 3C), suggesting that *Zfy2* plays a more dominant role in spermatogenesis than *Zfy1* and that a proper level of *Zfy1* and *Zfy2* gene expression is critical for spermiogenesis.

As antibodies to detect ZFY1 and ZFY2 are not available at this time, we could not completely exclude the possibility that partial peptides of ZFY1 or ZFY2 may contribute to our current findings. In addition, the role of each domain of ZFY1 and ZFY2 is still largely unknown. In future studies, using CRISPR/Cas9 to delete whole gene bodies or each functional domain will provide us detailed and solid information on the role of *Zfy1* and *Zfy2* in spermatogenesis.

Gene expression profiling in sperm is another challenge. Proteomic analysis, rather than transcriptome analysis, is well suited for analyzing expression profiles in mature sperm, because transcription is terminated [27]. Therefore, we carried out label-free proteomic analysis of *Zfy1/2*-DKO sperm and obtained candidates that could be responsible for the abnormal sperm formation.

The sperm-specific phospholipase C zeta 1, *Plcz1*, induces Ca^2+^ oscillations and activates metaphase II (MII) oocytes, resulting in completion of meiosis II [32]. In ICSI, if the oocyte fails to be activated and arrests at MII, the retained chromosome condensing factors should induce the PCC status of the injected sperm chromosomes [33]. Our results suggest that downregulated PLCZ1 expression in *Zfy1/2*-DKO sperm could decrease their oocyte-activating capacity and induce PCC, thus resulting in fertilization failure in ICSI.

Acrosomal exocytosis (also called the acrosome reaction) is a prerequisite for a sperm to fuse with an egg. Several genes are known to be involved in the acrosome reaction [26]. The acrosomal membrane protein, IZUMO, relocates from the acrosomal cap to the whole head region during the acrosome reaction [26]. In this study, we used IVF assay to show that the number of IZUMO-relocated sperm in *Zfy1/2*-DKO mutants was decreased compared to that in WT. *Plcd4* is required for calcium mobilization in the acrosome reaction [30], and *Prss21* is required for the acrosome reaction *in vitro* [29]. This suggests that downregulation of PLCD4 and PRSS21 in *Zfy1/2*-DKO sperm could cause a failure of the acrosome reaction in *Zfy1/2*-DKO sperm, in addition to a global absence of capacitation. As *Zfy1/2*-DKO sperm have impaired tyrosine phosphorylation, it is interesting that the functional annotation clustering analysis of both microarray and proteomic analysis showed several enriched GO terms that are involved in phosphorylation.

Elongating spermatids reduce nuclear volume through DNA compaction during spermiogenesis, and defects in DNA compaction can ultimately impact sperm nuclear morphology [34]. In this regard, deletion of the mouse ortholog of the human huntingtin gene, *Htt*, resulted in arrested spermatogenesis at the post-meiotic phase, partly due to disturbed translation and DNA packaging [31]. This suggests that the abnormal head phenotype of *Zfy1/2*-DKO sperm could be caused by a downregulation in HTT expression.

A wide variety of abnormalities in sperm deficient in both *Zfy1* and *Zfy2* could be due, at least in part, to decreased expression of PLCZ1, PLCD4, PRSS21, and HTT. In previous studies, *Zfy1* and *Zfy2* were considered transcription factors that activate target genes, raising the possibility that *Zfy1* and *Zfy2* directly regulate the expression of the above genes during spermatogenesis. However, it is difficult to examine this possibility because there is no available antibody that specifically recognizes ZFY1 and ZFY2, as mentioned above. Future studies using the insertion of an epitope tag sequence into the *Zfy1* and *Zfy2* genes using CRISPR/Cas9 will reveal whether *Zfy1* and *Zfy2* directly regulate these genes.

Artificial reproductive technology is conventionally performed for infertile couples throughout the world, and ICSI is performed for the male factor in infertile couples [32]. However, PLCZ deficiency and sperm DNA fragmentation are responsible for fertilization failure and poor embryo quality, respectively, and are an important issue in infertility patients [32]. Although mutations in *ZFY* have not been reported in infertile patients, our findings suggest that *ZFY* has a key role in spermatogenesis in humans.

In this study, we found that *Zfy1* and *Zfy2* are collectively indispensable for spermiogenesis. Elucidating the mechanisms that underlie *Zfy*-dependent spermatogenesis will shed light on the failure of fertilization and early embryonic development in mammals.

## Materials and Methods

### Ethics statement

The mice were anesthetized by intraperitoneal injection of pentobarbital. Cervical dislocation was used as a euthanasia method. All experiments were performed in accordance with the approved guidelines by the institutional committees for animal and recombinant DNA experiments at Tokyo Medical and Dental University. All animal experiments were approved by the Institutional Animal Care and Use Committee at Tokyo Medical and Dental University (approval number; 0170085A) [35].

### Generation of *Zfy1*-KO, *Zfy2*-KO, and *1/2*-DKO mice

The microinjection of mouse embryos was performed as described previously [11,36]. Mouse embryos were obtained either by mating superovulated BDF1 female mice and BDF1 male mice (Sankyo Lab Service) or as frozen BDF1 embryos (ARK Resource). The gRNA targets were designed for a common sequence in exon 4 of *Zfy1* and *Zfy2*. The target sequence of gRNA was 5ʹ-GAAGCAGTCTTAGATTCCAGTGG-3ʹ. The hCas9 and gRNA cloning vectors were prepared from Addgene (Cambridge, MA, USA). The gRNA expression vector was prepared by inverse PCR using following primers: fw 5ʹ-AAGCAGTCTTAGATTCCAGGTTTTAGAGCTAGAAATAGCA-3ʹ, rev 5ʹ-CTGGAATCTAAGACTGCTTCGGTGTTTCGTCCTTTCCACA-3ʹ. In order to obtain gRNA and hCas9 mRNA, *in vitro* transcription reactions were performed using the mMessage mMachine T7 Kit (Life Technologies) according to the manufacturer’s instructions. The template was prepared using the following primer: for hCas9, fw 5ʹ-TAATACGACTCACTATAGGGAGAATGGACAAGAAGTACTCCATTGG-3ʹ, rev 5ʹ-TCACACCTTCCTCTTCTTC-3ʹ; and for gRNA, fw 5ʹ-AAGGAATAATACGACTCACTATAGGGCTGCTCCAGAGGCATCCCAC-3ʹ, rev 5ʹ-TTTGAATTCGCACCGACTCGGTGCCACTT-3ʹ. The RNA purification was carried out using the MEGA Clear Kit (Life Technologies) according to the manufacturer’s instructions. The concentrations of injected gRNA and hCas9 mRNA were 250 ng/μl each. The RNA mixture was microinjected into the cytoplasm of the one-cell stage embryos. When frozen thawed embryos were used, we microinjected one-cell embryos 1 h after thawing (initial protocol) or 0.5 h after thawing (modified protocol). When fresh embryos prepared from superovulated female mice were used, we microinjected one-cell embryos 4 h after oocytes were retrieved (initial protocol), or 3 h after oocytes were retrieved (modified protocol). The embryos injected with RNAs were cultured in the M16 medium for one day and grown to the two-cell stage. They were then implanted in pseudopregnant ICR female mice.

### Genotyping

Genomic DNA was extracted from the tail tips of pups digested by Proteinase K. The genomic sequences around the gRNA target sites were PCR amplified using the following primers: for *Zfy1* ex4, fw 5ʹ-CCTACTCCAACCCACGTCAC-3ʹ, rev 5ʹ- TATCTTGCTGCTCCAGAGGC-3ʹ; for *Zfy2* ex4, fw 5ʹ- AGCTGATGCAGTACACATGGA-3ʹ, rev 5ʹ- TCATGATCCTCCCCTCCCTT-3ʹ. The PCR products were treated with ExoSAP-IT (USB) and sequenced directly [36].

### Off-target analysis

Putative off-target sites for gRNA were predicted by online software (http://crispr.mit.edu/) [23]. Three off-target sites were PCR amplified using the following primers and directly sequenced: for off-target 1, fw 5ʹ- AGACCACCAGTTCGTAGGTG-3ʹ, rev 5ʹ-ACAGCTTGATGCAAGGACTCG-3ʹ; for off-target 2, fw 5ʹ-TGCTCTGGCCCAGCTATAC-3ʹ, rev 5ʹ-AACATTTGAGCATGGCATGGAGTT-3ʹ; and for off-target 3, fw 5ʹ-GCATCACCTCAGGGGTTTGA-3ʹ, rev 5ʹ-CCCAGTGATTTCCCCAGTCC-3ʹ.

### Mating experiments

Fertility was investigated by mating 3-month-old BDF1, *Zfy1*-KO, *Zfy2*-KO, and *Zfy1/2*-DKO male mice with 8-week-old BDF1 female mice in one cage for two weeks; the animals were then separated from each other. We counted litter size after 3 weeks of separation. Pregnancy success was calculated as the number of litters produced/number of attempted breedings [37]. For BDF1 female mice that did not give birth, we confirmed whether their fertility was normal by mating them with BDF1 male mice.

### Quantitative real-time PCR assay

Total RNAs were prepared from 1-week-old to 5-week-old BDF1 testes using the ReliaPrep RNA Tissue Miniprep System (Promega). A mixture of the homogenized testes of three mice of each age was analyzed. Quantitative gene expression analysis was performed using THUNDERBIRD SYBR qPCR Mix on a Thermal Cycler Dice Real Time System II (TAKARA). Data were normalized to GAPDH gene expression for each experiment. The primer sequences are shown below.

For *Zfy1*; fw 5ʹ-CAGGCATTCTGGGAACGGAA-3ʹ, rev 5ʹ-TACTGGGCCGGTCTCTTACA-3ʹ.

For *Zfy2*; fw 5ʹ-CGCCACCAAAGCCAAAAGAT-3ʹ, rev 5ʹ-GCCGGTCTCTGGCTTTAATGTAT-3ʹ.

For *GAPDH*; fw 5ʹ-CCTGGTCACCAGGGCTGC-3ʹ, rev 5ʹ-CGCTCCTGGAAGATGGTGATG-3ʹ.

### Sperm count

The testis and epididymis were cleared of adhering tissues and homogenized (AM. 2030, BiomasherI, SARSTEDT) in phosphate buffer saline, 0.5% Triton X-100 (Wako). Sperm were counted in aliquots of diluted homogenate using a hemocytometer [38].

### HE and PAS-haematoxylin staining

Bouin’s solution-fixed, paraffin-embedded testes and epididymides were sliced into 8-μm-thick sections. The slices were stained with haematoxylin-eosin [11]or periodic acid schiff (PAS)-haematoxylin. Sperm were retrieved from the cauda epididymis and suspended in phosphate-buffered saline, and the suspension was smeared on a slide.

### Transmission electron microscopy

The specimens were fixed with 2.5% glutaraldehyde in 0.1 M phosphate buffer (PB) for 2 h. They were washed with 0.1 M PB, post-fixed in 1% OsO_4_ buffered with 0.1 M PB for 2 h, dehydrated in a graded series of ethanol, and embedded in Epon 812. Ultrathin (90 nm) sections were collected on copper grids, double-stained with uranyl acetate and lead citrate, and then observed using transmission electron microscopy (H-7100, Hitachi, Tokyo, Japan).

### Immunohistochemical observation of the testes and epididymal sperm

For immunohistochemical examination, testes were fixed in Bouin’s or 4% paraformaldehyde solution, embedded in paraffin, and sectioned at a thickness of 8 μm. Deparaffinized sections were incubated with MitoTracker Red CM-H2Xros (Thermo Fisher Scientific, Yokohama, Japan), FITC-peanut agglutinin (Sigma-Aldrich, Tokyo, Japan), or anti-MCA rabbit antibody[39,40], as in the previous study. Sperm from the cauda epididymis were cultured in PBS for 10 min, spotted onto glass slides, and dried. Sections were treated with MitoTracker Red CM-H2Xros, anti-protamine monoclonal antibodies [39], anti-MCA [40], Shippo1/Odf3 [41], or Tektin-t/Tekt2 [42] polyclonal antibodies as in the previous studies and counterstained with DAPI (Nacalai Tesque, Kyoto, Japan) or propidium iodide (Sigma-Aldrich). The slides were washed and examined under a fluorescent microscope.

### Analysis of sperm motility

Overall sperm motility was assessed using a Hamilton Thorn IVOS computerized semen analyzer (Hamilton Thorn, Beverly, MA). Motility was defined as any movement of the sperm head. Overall sperm motility was measured for 300 spermatozoa in at least three different fields.

### IVF

Sperm were retrieved from the cauda epididymis of male mice and capacitated in HTF medium (ARK Resource) at 37°C for 1 h. Mature oocytes were retrieved from oviducts of superovulated BDF1 female mice. Cumulus oocyte complexes were placed in a 200-μl drop of HTF medium, and were co-incubated (37°C, 5% CO_2_) with 1 × 10^5^/mL capacitated sperm.

After 2 h insemination, oocytes were washed with KSOM and incubated in KSOM for 4 h. After 6 h insemination, we counted oocytes with two or more pronuclei as fertilized oocytes.

### Acrosome reaction analysis

Sperm and oocytes were retrieved as described above. Sperm were capacitated in HTF medium (ARK Resource) at 37°C for 1 h. Sperm were collected 2 h after insemination with cumulus oocyte complex, smeared on a slide glass, and air-dried. Control sperm were collected an additional 2 h after incubation with HTF medium. Sperm were fixed with ethanol at -20°C for 10 min and incubated with 10% FBS at RT for 30 min, incubated with anti-IZUMO antibody (1:200, 73–045, B-bridge) at RT for 1 h, and then incubated with Goat anti-Rat IgG (H+L) secondary antibody, Alexa Fluor 488 conjugate (Thermo Fisher, A-11006). VECTASHIELD Mounting Medium with DAPI (Vector Laboratories, H-1200) was used for nuclear counterstaining. We counted sperm stained only in the acrosomal cap as acrosome unreacted, and sperm stained in the equatorial or whole head as acrosome reacted [25]. We counted at least 100 sperm.

### ICSI

Six- to 10-week-old BDF1 and 8- to 12-week-old ICR female mice were used for oocyte collection and as embryo transfer recipients, respectively. BDF1 females were injected intraperitoneally with 7.5 units of equine chorionic gonadotropin (Teikoku-Zoki Pharmaceuticals, Tokyo, Japan), followed by an injection of 7.5 units of human chorionic gonadotropin (hCG; Aska Pharmaceuticals, Tokyo, Japan) 48–50 h later. Mature oocytes were collected from oviducts at 15–17 h after hCG injection and were freed from cumulus cells by 1 min treatment with 0.1% hyaluronidase (Sigma-Aldrich, St. Louis, MO, U.S.A.) in CZB medium. The oocytes were transferred to fresh CZB medium and incubated at 37°C in an atmosphere of 5% CO_2_ until they were used for microinjection.

ICSI experiments were performed using a micropipette attached to a piezo-electric actuator (PrimeTech, Ibaraki, Japan), as previously described [43,44]. Briefly, epididymal spermatozoa obtained from the cauda epididymides of donor males were dispersed in CZB medium. Spermatozoa were placed in a Hepes-CZB containing 10% polyvinylpyrrolidone. A single spermatozoa was sucked, tail first, into an injection pipette and the head was separated from the tail by applying a few piezo pulses to the head–tail junction. The isolated sperm head was injected into an oocyte in Hepes-CZB. The injected oocytes were then kept in Hepes-CZB at room temperature (25°C) for 10 min before being cultured in CZB at 37°C under 5% CO2 in air. Embryos that reached the two-cell stage by 24 h were transferred into the oviducts of pseudopregnant ICR females the day after sterile mating with a vasectomized male (day 0.5). On day 19.5, the recipient mice were sacrificed and their uteri were examined for the presence of live term fetuses. Some remaining embryos were cultured for 96 h to determine the rate of development to blastocysts.

### Examination of oocytes

Sperm-injected oocytes that failed to develop to the two-cell stage were examined by a whole-mount preparation method as previously reported [45]. Briefly, oocytes were mounted and gently compressed between a slide glass and a coverslip. They were fixed with 2.5% glutaraldehyde in cacodylate buffer, washed with water, and dehydrated with 100% ethanol. After being stained with 1% acetoorceine in 45% acetic acid, oocytes were examined with a phase-contrast microscope.

### Chromosome preparation

For chromosome preparation experiments, intact or pre-enucleated oocytes were used for ICSI. At 6 to 8 h after ICSI, oocytes with a pronucleus (or pronuclei) were transferred into CZB medium containing 0.003 μg/mL vinblastine sulfate salt (SIGMA-Aldrich) and cultured until they were arrested at the metaphase of the first cleavage. At 18 to 21 h after ICSI, the mitotic embryos were treated with 0.5% pronase for 5 min to remove the zona pellucida. Zona-free eggs were then transferred in hypotonic solution (1:1 mixture of 1% sodium citrate and 60% FCS) for 10 min at room temperature. Chromosome spreads were prepared on slide glasses by the gradual fixation/air drying method [46]. The slides were stained with 5% Giemsa in PBS (pH 6.8) for 8–10 min.

### Microarray analysis

Total RNAs were prepared from testes using the ReliaPrep RNA Tissue Miniprep System (Promega). They were reverse-transcribed into cDNA with ReverTra Ace (TOYOBO). Labeling and hybridization of samples was performed via the GeneChip 3’IVT Expression Kit (Affymetrix) and GeneChip Mouse Genome 430 2.0 Array (Affymetrix), respectively, according to the manufacturer’s instructions. The data were normalized by the robust multichip analysis (RMA) procedure. The data was analyzed using DAVID (https://david.ncifcrf.gov/).

### Mass spectrometric protein profiling

The protein samples (4.5 μg) were separated by SDS-PAGE (12.5% acrylamide gel; ATTO, Tokyo, Japan). Each gel lane was cut into 48 pieces of equal size using a GridCutter (Gel Company, San Francisco, CA, USA), and each gel piece was subjected to in-gel tryptic digestion as described previously [47]. All MS experiments were performed on a Finnigan LTQ Orbitrap XL mass spectrometer (Thermo Fisher Scientific, MA, USA) equipped with a nanoelectrospray ion source (AMR, Tokyo, Japan). In brief, the tryptic digests were injected to a nanoflow HPLC system (AMR, Tokyo, Japan) connected to the mass spectrometer. The peptides were separated in a 15-cm length analytical column (100-μm inner diameter) with 3-μm C18 beads (AMR, Japan). The separation was carried out at a flow of 250 nl/min, using 0.1% aqueous formic acid solution as eluent A and 0.1% formic acid in 90% aqueous acetonitrile solution as eluent B. A 140-min gradient from 5% to 45% acetonitrile was performed. The separated peptides were electrosprayed into the mass spectrometer.

A spray voltage of 1.8 kV was applied to ionize the peptides. Full MS scans were performed using an orbitrap mass analyzer. The five most intense precursor ions were selected for the MS/MS scans, which were performed using collision-induced dissociation (CID) for each precursor ion. For survey scans (mass range, 350–1,800 m/z), the target value was 5e+05 with a maximum injection time of 500 ms and a resolution of 60,000 at m/z 400. An MS/MS isolation window of 2.0 m/z was used for CID with normalized collision energies of 35. For MS/MS scans, the target ion value was set to 1e+04 with a maximum injection time of 100 ms and dynamic exclusion of 60 s.

The Mascot software package (version 2.5.1; Matrix Science, London, U.K.) was used to identify the mass of each peptide ion peak against the SWISS-PROT database (*Mus musculus*, 16777 sequences in the Swiss prot_2015_09.fasta file) using the following parameters: 1 missed cleavage; variable modifications: oxidation (Met); peptide tolerance: 20 ppm; and MS/MS tolerance: 0.8 Da; peptide charge: 2+ and 3+. To evaluate the relative protein abundance, the exponentially modified Protein Abundance Index (emPAI) was employed [48]. When the ratio of emPAI score between *Zfy1/2*-DKO and WT was less than 0.5, we considered the difference significant. The functional annotation clustering analysis was performed using DAVID online software.

### Western blot analysis

Sperm were collected from the cauda epididymis in PBS, suspended in RIPA buffer (50 mM Tris-HCl pH 7.4, 150 mM NaCl, 1% NP-40, 1% sodium deoxycholate, 1% SDS, 0.5 mM PMSF), and incubated at 4°C for 30 min. After centrifugation, the supernatant was collected, and the soluble protein was measured using the DC Protein Assay Kit (Bio-Rad, Hercules, CA, USA). Sperm protein samples (20 μg/lane) were subjected to protein separation by SDS-polyacrylamide gel electrophoresis; separated proteins were transferred to polyvinylidene difluoride membranes. Membranes were incubated with Blocking One (Nacalai Tesque) for 1 h; incubated with PLCZ1-antibody (1:1000, ab124446, Abcam), HTT-antibody (1:5000, ab109115, Abcam), PRSS21-antibody (1:400, AF6820, R&D), PLCD4-antibody (sc-30063, Santa Cruz), α/β tubulin antibody (1:1000, 2148S, Cell Signaling Technology) or β-actin antibody (1:2000, A5316, Sigma) at 4°C overnight, and then incubated for 1 h with HRP-conjugated anti-rabbit IgG antibody (1:10,000, A6154, Sigma) or HRP-conjugated anti-mouse IgG antibody (1:10,000, A2304, Sigma). Proteins were detected by Pierce ECL Western Blotting Substrate (32106, Thermo Fisher Scientific) or ECL Select Western Blotting Detection Reagent (RPN2235, GE Healthcare).

In order to detect tyrosine phosphorylation of the sperm proteins, the sperm were incubated at 37°C with HTF medium in an atmosphere that contained 5% CO_2_ [24]. After incubation, the sperm were collected and washed with PBS and then dissolved in RIPA buffer containing 1mM sodium orthovanadate (NEW ENGLAND BioLabs). Sperm protein samples (10 μg/lane) were subjected to protein separation and transferred to membranes as described above. Membranes were incubated Blocking One (Nacalai Tesque) for 1 h; incubated with phosphotyrosine antibody (1:1000, clone4G10, Merck Millipore) at 4°C for overnight; and incubated for 1 h with HRP-conjugated anti-rabbit IgG antibody (1:10,000, A6154, Sigma). Phosphotyrosine signals were visualized as described above.

## Supporting Information

S1 Fig Sequences around *Zfy1* and *Zfy2* targets of *Zfy1* knockout (KO) mice, *Zfy2*-KO mice, and *Zfy1* and *Zfy2* double knockout (*Zfy1/2*-DKO) mice. PAM sequences are shown in red. Inserted sequences are shown in blue.
(TIF)

S2 Fig Electropherograms around the sequence targeted by guide RNA in WT and *Zfy1* and *Zfy2* double knockout (*Zfy1/2*-DKO) mice. (TIF)

S3 Fig Age-related *Zfy1* and *Zfy2* expression in the testis. Quantitative real-time PCR analysis of testes of 1- to 5-week-old BDF1 male mice. GAPDH was used as an internal control. The error bars represent the standard deviation of triplicates. dpp; days post partum.
(TIF)

S4 Fig Immunohistological analyses of mutant sperm and testes. Epididymal sperm of wild-type (WT), *Zfy2* knockout (KO), and *Zfy1* and *Zfy2* double knockout (*Zfy1/2*-DKO) mice were separated on the glass slide and examined under a fluorescent microscope (A, B). (A) Immunohistochemical observations of mutant sperm flagella. The major flagellar components of MCA, ODF3, and TEKT2 were localized in mutant sperm flagella without serious differences from the wild type. (B) Nuclear localization of PRM 1 and 2. PRMs were localized in the mutant sperm nuclei, although nuclear formation was abnormal. (C) Immunohistochemical observations of mutant testicular cross-sections. The acrosome, flagellum, and mitochondrion were observed with peanut agglutinin (PNA) (Bouin's fixation), MCA (PFA), and MitoTracker (Mit) (Bouin's fixation), respectively. Morphological abnormality was not found in mutant testes. Non-specific signals were observed on Leydig cells in MCA panels.
(TIF)

S5 Fig Chromosome spread at first mitosis. Normal chromosomal spread of *Zfy1* and *Zfy2* double knockout sperm ICSI-derived embryo at the first mitosis.
(TIF)

S1 Table Putative off-target sites of gRNA. (PDF)

S2 Table Downregulated probe ID in microarray analysis. (XLSX)

S3 Table Enriched GO terms in microarray analysis. (XLSX)

S4 Table 3191 proteins identified in WT sperm. (XLSX)

S5 Table 3766 proteins identified in Zfy1/2-DKO sperm. (XLSX)

S6 Table Downregulated proteins in *Zfy1/2*-DKO sperm. (XLSX)

S7 Table Enriched GO terms in proteomic analysis. (XLSX)

S8 Table Downregulated proteins and those annotated with sexual reproduction in *Zfy1/2*-DKO sperm. (XLSX)
