# Supplementary figures and images for: Complementary Critical Functions of Zfy1 and Zfy2 in Mouse Spermatogenesis and Reproduction
Source: PLoS Genet. 2017 Jan 23;13(1):e1006578. doi: 10.1371/journal.pgen.1006578 (PMC5287576; doi:10.1371/journal.pgen.1006578)

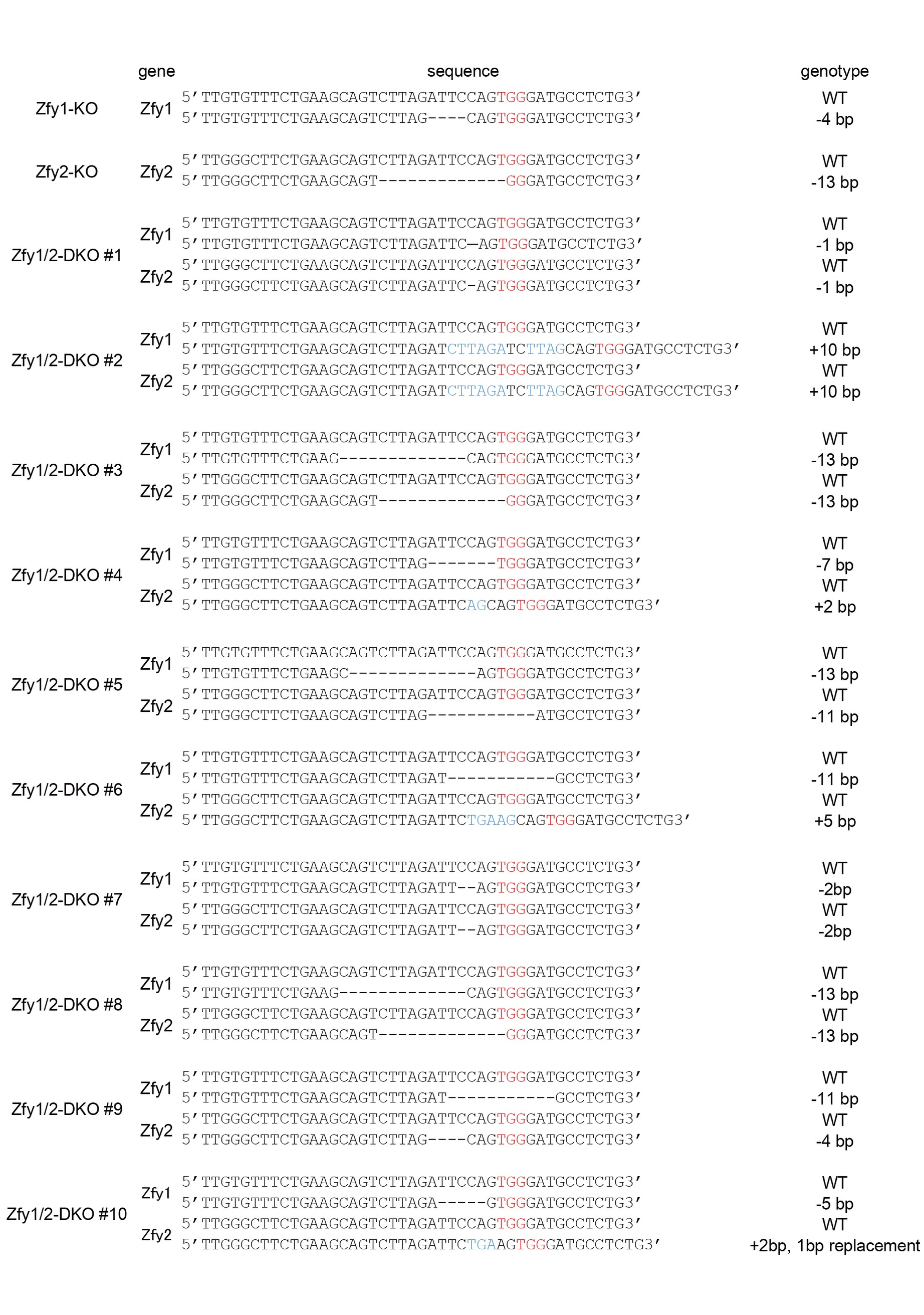

Supplement: S1 Fig — PAM sequences are shown in red. Inserted sequences are shown in blue. (TIF) [file pgen.1006578.s001.tif]

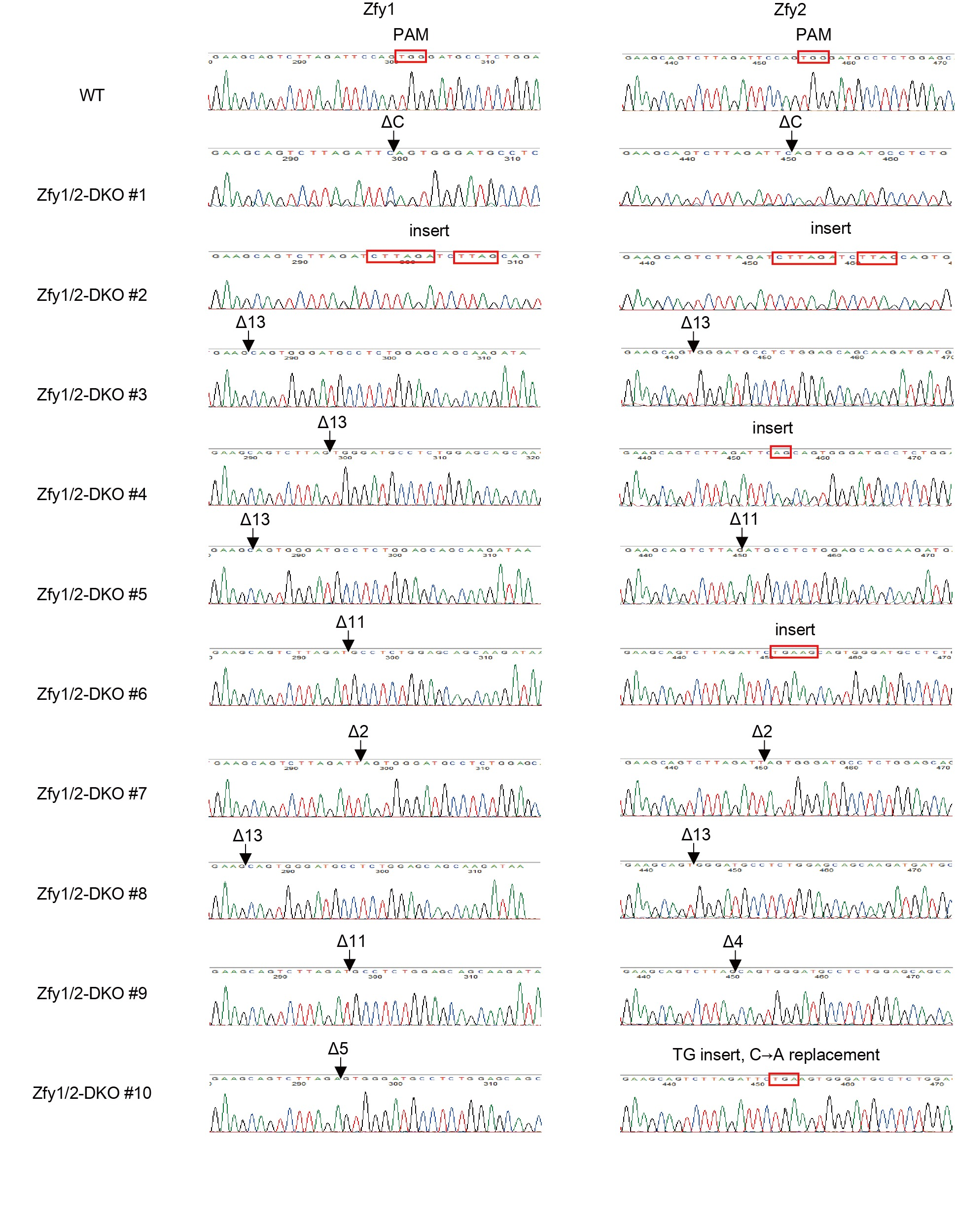

Supplement: S2 Fig — (TIF) [file pgen.1006578.s002.tif]

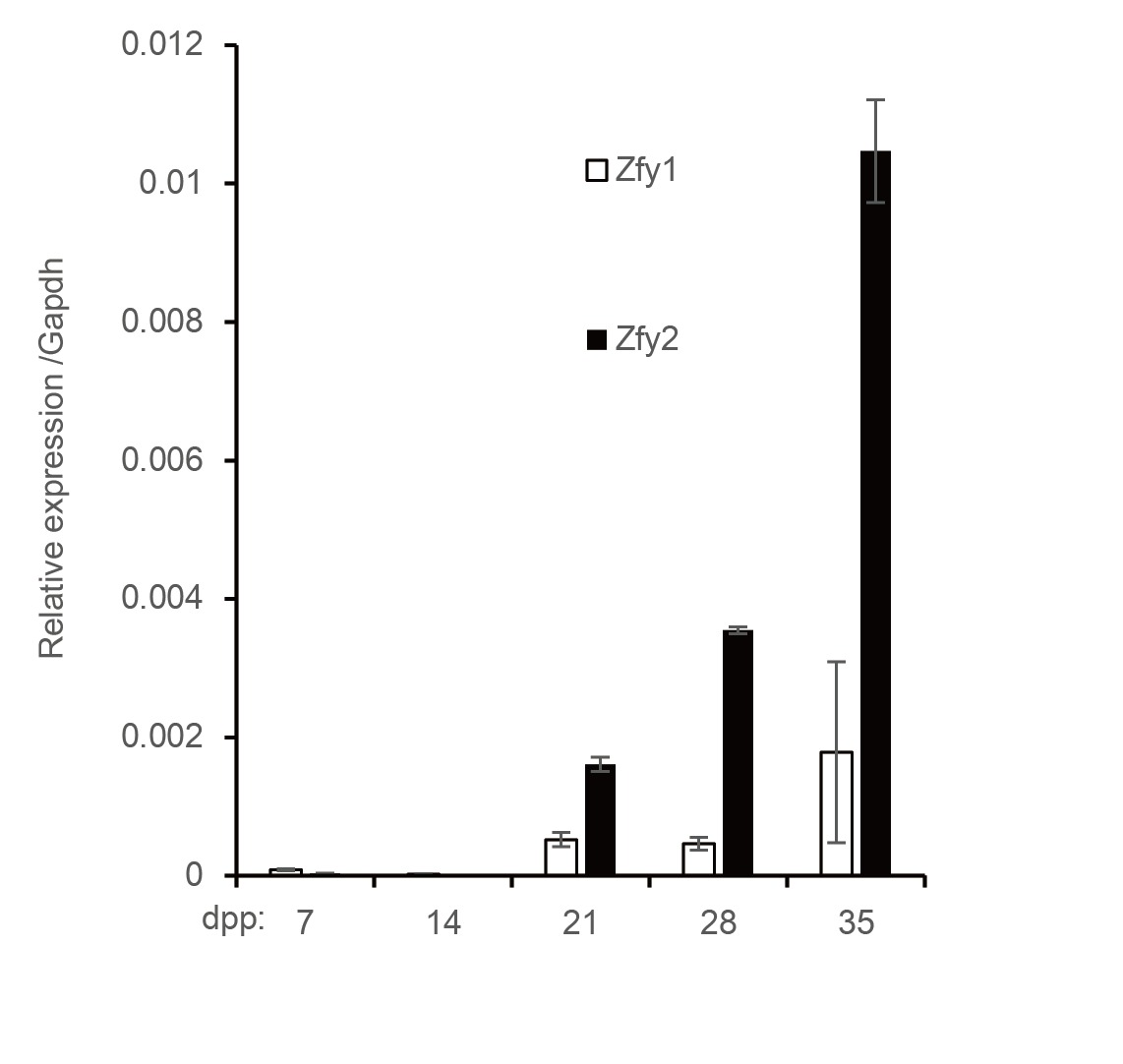

Supplement: S3 Fig — Quantitative real-time PCR analysis of testes of 1- to 5-week-old BDF1 male mice. GAPDH was used as an internal control. The error bars represent the standard deviation of triplicates. dpp; days post partum. (TIF) [file pgen.1006578.s003.tif]

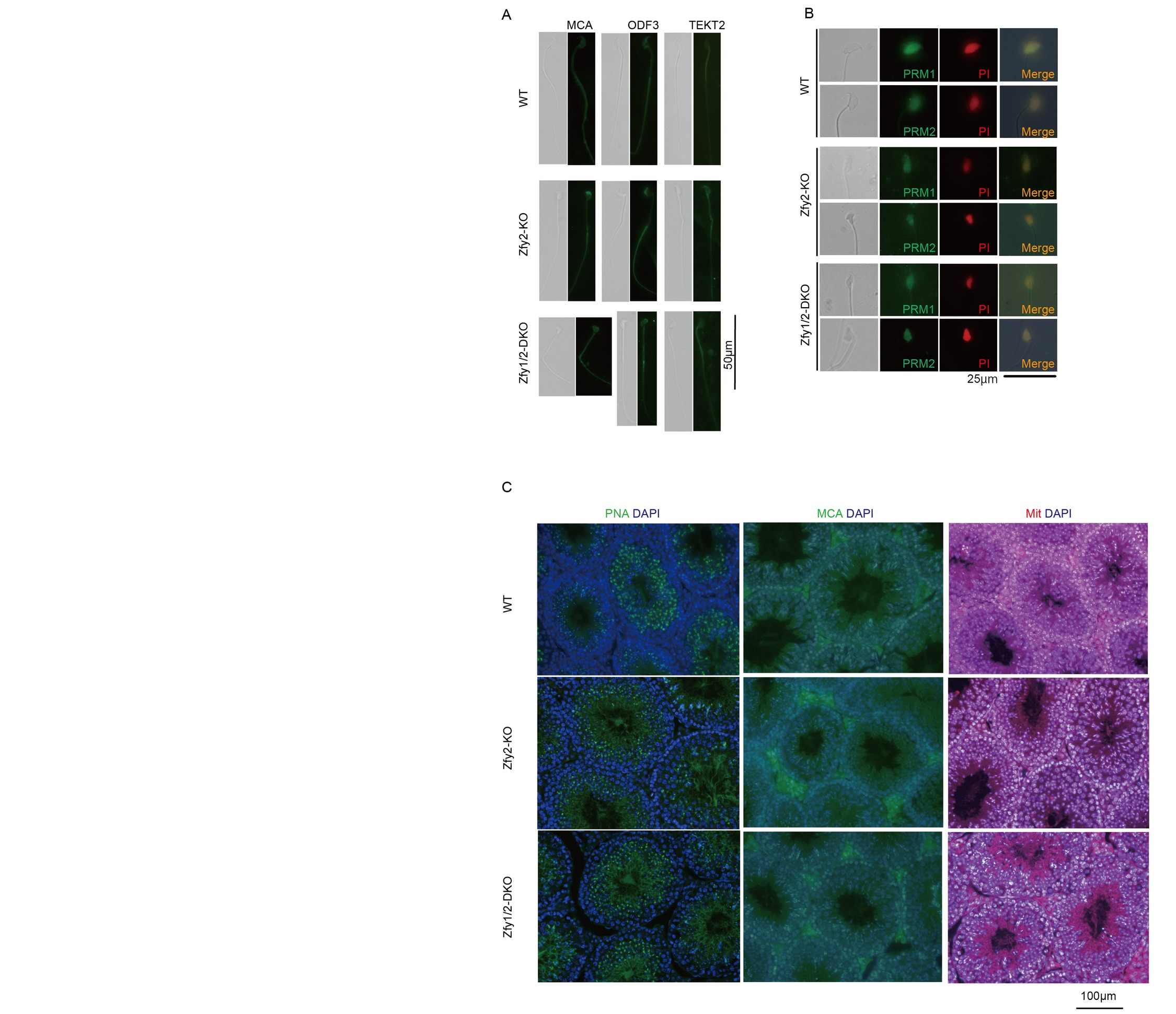

Supplement: S4 Fig — Epididymal sperm of wild-type (WT), Zfy2 knockout (KO), and Zfy1 and Zfy2 double knockout (Zfy1/2-DKO) mice were separated on the glass slide and examined under a fluorescent microscope (A, B). (A) Immunohistochemical observations of mutant sperm flagella. The major flagellar components of MCA, ODF3, and TEKT2 were localized in mutant sperm flagella without serious differences from the wild type. (B) Nuclear localization of PRM 1 and 2. PRMs were localized in the mutant sperm nuclei, although nuclear formation was abnormal. (C) Immunohistochemical observations of mutant testicular cross-sections. The acrosome, flagellum, and mitochondrion were observed with peanut agglutinin (PNA) (Bouin's fixation), MCA (PFA), and MitoTracker (Mit) (Bouin's fixation), respectively. Morphological abnormality was not found in mutant testes. Non-specific signals were observed on Leydig cells in MCA panels. (TIF) [file pgen.1006578.s004.tif]

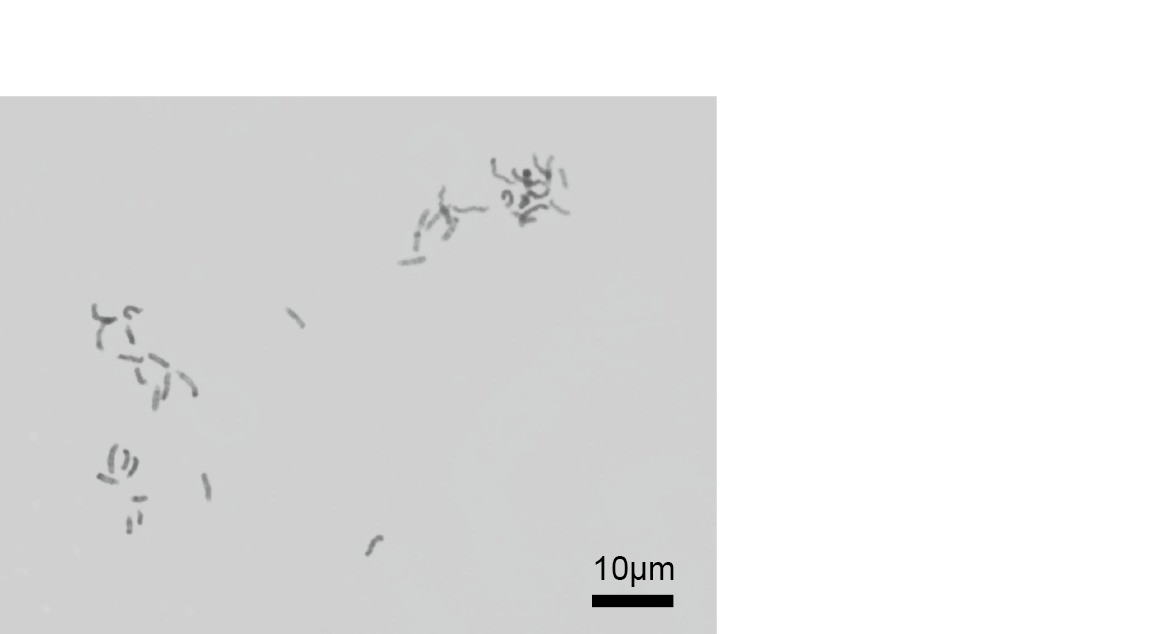

Supplement: S5 Fig — Normal chromosomal spread of Zfy1 and Zfy2 double knockout sperm ICSI-derived embryo at the first mitosis. (TIF) [file pgen.1006578.s005.tif]
