# Supplementary material for: Complementary Critical Functions of Zfy1 and Zfy2 in Mouse Spermatogenesis and Reproduction
Source: PLoS Genet. 2017 Jan 23;13(1):e1006578. doi: 10.1371/journal.pgen.1006578 (PMC5287576; doi:10.1371/journal.pgen.1006578)
Supplement: S1 Table — (PDF) [file pgen.1006578.s006.pdf]

Supplementary Table 1 Putative off-target sites of gRNA.

	chromosome	position	strand	sequence	mismatches
Zfy1	chrY	75092	-	GAAGCAGTCTTAGATTCCAGTGG	0
Zfy2	chrY	1377284	-	GAAGCAGTCTTAGATTCCAGTGG	0
OT-1	chr2	8963046	+	GAAGCAGTCTTACATTCCAGTAG	1
OT-2	chr9	96980720	+	GAAGCAGTCTTACATTCCAGTAG	1
OT-3	chrX	98614155	+	GGGGCAGTCTGAGATTCCAGCAG	3

OT, off-target site. PAM sequences are shown in red. Mismatches are shown in blue
